# Intercalated Iron Chalcogenides: Phase Separation Phenomena and Superconducting Properties

**DOI:** 10.3389/fchem.2021.640361

**Published:** 2021-06-22

**Authors:** Anna Krzton-Maziopa

**Affiliations:** Faculty of Chemistry, Warsaw University of Technology, Warsaw, Poland

**Keywords:** layered superconductors, intercalated iron chalcogenides, superconductivity, crystal structure, phase separation

## Abstract

Organic molecule-intercalated layered iron-based monochalcogenides are presently the subject of intense research studies due to the linkage of their fascinating magnetic and superconducting properties to the chemical nature of guests present in the structure. Iron chalcogenides have the ability to host various organic species (i.e., solvates of alkali metals and the selected Lewis bases or long-chain alkylammonium cations) between the weakly bound inorganic layers, which opens up the possibility for fine tuning the magnetic and electrical properties of the intercalated phases by controlling both the doping level and the type/shape and orientation of the organic molecules. In recent years, significant progress has been made in the field of intercalation chemistry, expanding the gallery of intercalated superconductors with new hybrid inorganic–organic phases characterized by transition temperatures to a superconducting state as high as 46 K. A typical synthetic approach involves the low-temperature intercalation of layered precursors in the presence of liquid amines, and other methods, such as electrochemical intercalation, intercalant or ion exchange, and direct solvothermal growths from anhydrous amine-based media, are also being developed. Large organic guests, while entering a layered structure on intercalation, push off the inorganic slabs and modify the geometry of their internal building blocks (edge-sharing iron chalcogenide tetrahedrons) through chemical pressure. The chemical nature and orientation of organic molecules between the inorganic layers play an important role in structural modification and may serve as a tool for the alteration of the superconducting properties. A variety of donor species well-matched with the selected alkali metals enables the adjustment of electron doping in a host structure offering a broad range of new materials with tunable electric and magnetic properties. In this review, the main aspects of intercalation chemistry are discussed, involving the influence of the chemical and electrochemical nature of intercalating species on the crystal structure and critical issues related to the superconducting properties of the hybrid inorganic–organic phases. Mutual relations between the host and organic guests lead to a specific ordering of molecular species between the host layers, and their effect on the electronic structure of the host will be also argued. A brief description of a critical assessment of the association of the most effective chemical and electrochemical methods, which lead to the preparation of nanosized/microsized powders and single crystals of molecularly intercalated phases, with the ease of preparation of phase pure materials, crystal sizes, and the morphology of final products is given together with a discussion of the stability of the intercalated materials connected with the volatility of organic solvents and a possible degradation of host materials.

## Introduction

Discovery of superconductivity in a non-stoichiometric binary iron selenide compound FeSe_1−*x*_ (Hsu et al., 2008) and shortly after in its ternary analogs: FeSe_1−*x*−*y*_*Ch*_*y*_ (*Ch* = S, Te) with isovalent substitutions on the selenium site (Fang et al., 2008; Yeh et al., 2008; Mizuguchi et al., 2009a), widened the gallery of layered superconductors. Research on the effects of chemical modification, i.e., the substitution of an isovalent anion on critical temperature, intertwined with the studies of hydrostatic pressure effects resulted in a multitude of reports combining distortions in the crystal structure of parent compounds with the critical temperature of superconducting transition. Another peculiar feature of these systems is the high susceptibility of the crystal structure to hydrostatic pressure that affects the geometry of the iron-selenium tetrahedron. In all these groups, the application of pressure (either hydrostatic or chemical) results in a significant increase in *T*_c._ For example, in FeSe_1−*x*_ that crystallizes in a tetragonal structure, on increase of hydrostatic pressure from atmospheric pressure to the value of about 7.5 GPa, the critical temperature of superconductivity raises from about 8.5 to 36.5 K. These effects have been usually attributed to the strain-driven modifications of the FeSe_4_ tetrahedron geometry. The tetrahedron is distorted and the Se-Fe-Se angles deviate significantly from the tetrahedral ones at normal pressure, while the action of external hydrostatic pressure forces the geometrical modification of the deformed tetrahedrons to greater regularity (Mizuguchi et al., 2008; Margadonna et al., 2009; Medvedev et al., 2009). It can be hypothetically assumed that more regular geometry should result in higher critical temperatures although this is only a rough assumption, which ignores other important factors, such as the injection of charge carriers resulting from the presence of structural defects, vacancies, interstitial atoms, or inclusions that may serve as doping centers. Considering a consequence of the application of hydrostatic pressure on non-stoichiometric iron selenide, it was expected that alike effects would appear on chemical substitutions in the selenium sublattice with tellurium or sulfur in the layered FeSe_1−*x*_, due to the creation of internal “chemical pressure” being the result of the mismatch of ionic radii. Indeed, the experiments carried out on the ternary phases FeSe_(1−*x*−*y*)_*Ch*_*y*_ (*Ch* = S or Te) showed the increase of *T*_c_ with the retention of a tetragonal structure to a certain level of substitution in the anion sublattice, but the observed effects on the critical temperature were much less than expected. Notably, at an optimum sulfur concentration (*y* ~ 0.2), an increase in *T*_c_ was about 10.5 K, and for tellurium (*y* ~ 0.5) was about 15.5 K (Fang et al., 2008; Yeh et al., 2008; Mizuguchi et al., 2009a,b). Nevertheless, this allowed for a general assumption that subtle structural modifications create an opportunity for fine tuning of the critical temperature of transition to a superconducting state in these systems (Margadonna et al., 2009; Tsoi et al., 2009; Mizuguchi et al., 2010; Okabe et al., 2010). The chemical pressure exerted either by sulfur or tellurium substitutions in the anion sublattice was possibly not high enough to achieve the higher *T*_c_ values.

**Figure 1:**
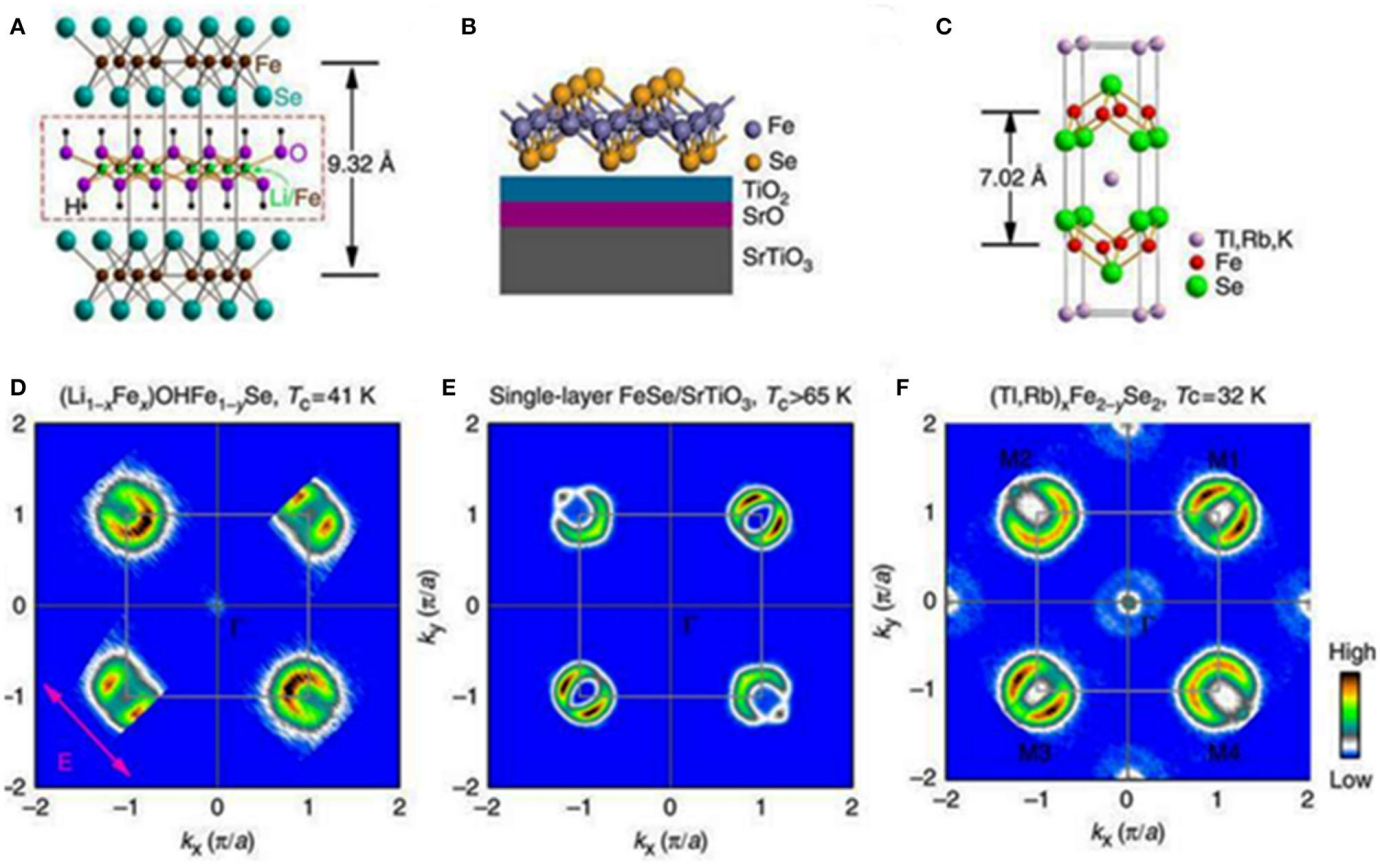
Models of crystal structure and similarities of the Fermi surface topology of three Fe-based superconductors **(A)** The crystal structure of (Li_1−*x*_Fe_*x*_)OHFe_1−*y*_Se, **(B)** schematic structure of single-layer FeSe film deposited on a SrTiO_3_ substrate, **(C)** schematic structure of (Tl,Rb)_*x*_Fe_2−*y*_Se_2_, **(D–F)** fermi surface maps of corresponding superconductors. [Reprinted with permission from Zhao et al. (2016) Copyright (2016) Nature Publishing Group].

Although binary and ternary iron chalcogenides did not show impressive values of transition temperatures to a superconducting state, which typically range from 8.5 K for FeSe_0.98_ to about 15.5 K for FeSe_0.5_Te_0.5_, the phenomenon of superconductivity occurring in these materials along with the stripy nematic-like ordering of magnetic domains coexisting with superconducting properties intrigued scientists and caused an immediate increase in the number of publications within a few months. A specific layered arrangement of iron atoms in the cation sublattice enforces two-dimensionality of electronic structure in which common energy bands, composed of overlapping Fe 3*d* and Se 4*p* orbitals, are formed in close proximity to the Fermi level. The accurate geometry of the energy bands and topology of the Fermi surface are closely related to the chemical composition and crystal structure (Johnston, 2010; Stewart, 2011; Glasbrenner et al., 2015; Liu et al., 2015). This implies also other electronic properties of these materials, which likewise most of the pristine phases and also underdoped iron-based pnictides reveal the presence of a nematic-like long-range antiferromagnetic order (Fernandes et al., 2014). A common feature of these materials is the emergence of superconductivity on the suppression of magnetic order either by the pressure-induced suppression of nematicity or delicate structural changes caused by chemical doping (Dai et al., 2012; Wang et al., 2016). Regardless of the structural similarity of FeSe_1−*x*_ and Fe_1+δ_Te, their low-temperature behavior is completely different. On cooling to temperatures lower than 90 K, the tetragonal structure of FeSe_1−*x*_ transforms into an orthorhombic one, and the material becomes superconducting below 8.5 K (Hsu et al., 2008; McQueen et al., 2009), whereas in Fe_1+δ_Te the antiferromagnetic commensurate order, driven by a structural transition from tetragonal phase to monoclinic phase, appears below 70 K (Bao et al., 2009; Li et al., 2009; Jorgensen and Hansen, 2015). Few studies on the electronic structure of FeSe_1−*x*_ with angle-resolved photoemission spectroscopy (ARPES) revealed the discrepancies between the experimental band structure and theoretical predictions based on density functional theory calculations (Subedi et al., 2008). It was found that considerable orbital-dependent changes start below the temperature related to the orthorhombic distortion, and continue further on cooling to the transition to a superconducting state (Maletz et al., 2014). Partial substitution of tellurium with other chalcogens results in a gradual decay of antiferromagnetic order bringing superconducting Fe_1+δ_Te_1−*x*_Se_*x*_ phases with maximum *T*_c_ = 14 K for *x* = 0.5 at ambient pressure (Fang et al., 2008; Yeh et al., 2008; Liu et al., 2011). In Fe_1+δ_Te_1−*x*_Se_*x*_ tetragonal phases, the positive effect of hydrostatic pressure on the superconducting properties has also been noted, although the observed changes were smaller in comparison to the tetragonal iron selenide. Few studies on the dependence of *T*_c_ on the applied pressure performed on Fe_1.03_Te_0.43_Se_0.57_ have shown that the critical temperature of superconductivity initially increased slowly with the pressure at 3.1 GPa up to maximum *T*_c_ = 23.2 K. Higher values of pressure resulted in a gradual decrease of *T*_c_ accompanied by its complete suppression above 11.9 GPa. These findings were correlated with the structural changes studied using high-resolution synchrotron x-ray diffraction, which evidenced that the orthorhombic phase, attributed to superconductivity below 40 K, occurs at ambient pressure and begins to transform into a non-superconducting monoclinic one already at pressures above 3 GPa (Gresty et al., 2009). All the above points support the conviction about the correlations between the crystal structure and electronic properties in Fe-based chalcogenide superconductors. Moreover, the superconducting properties of these materials are strongly anisotropic as illustrated in the results of transport and magnetic investigations performed on single crystals of Fe_1+δ_Te_1−*x*_Se_*x*_ (Yadav and Paulose, 2009; Biswas et al., 2010; Mook et al., 2010; Glasbrenner et al., 2015). Although the topology of the Fermi surface of all the tetragonal monochalcogenides (FeSe, FeS, and FeTe) is similar to that of iron pnictides, with a characteristic cylindrical hole in the center and electron pockets located in the corners of the Brillouin zone (Maletz et al., 2014), it does not imply the same electric or magnetic behavior. In these materials, there are other, not yet fully understood mechanisms standing behind the charge transfer processes. Reports on surprisingly high critical temperatures above 70 K observed in the monolayers of Fese_1−*x*_ grown on diverse substrates (Wang et al., 2012; Sun et al., 2014; Kang and Fernandes, 2016; Zhao et al., 2016), support these assumptions, though the Fermi surface topology observed for the FeSe monolayers (only electron pockets) differs significantly from the bulk materials, and what is more interesting, both long-range magnetic order and nematicity are absent in monolayer films (Liu et al., 2012; He et al., 2013). The magnetic and electronic properties of thin films depend strongly on their thickness: when the film is thicker than a few unit cells, its electronic structure is similar to that of a bulk material and is usually not superconducting with a well-pronounced nematic order (Zhang et al., 2016; Li et al., 2017). The critical temperature of the FeSe monolayers is in fact the highest among all iron-based superconductors known so far, hence the answer to the questions about its origin would be an important discovery in a search for high critical temperatures in iron-based superconductors. There are a few provisional hypotheses proposed for an explanation of the observed enormous enhancement of *T*_c_ in the FeSe monolayers, involving an enhanced coupling to the optical phonon mode of the substrate (Liu et al., 2012; Wang et al., 2012; He et al., 2013; Sun et al., 2014; Kang and Fernandes, 2016). Although the proposed mechanism was supported by ARPES studies (Tan et al., 2016), it fits well only for the strontium titanate substrate and does not explain the origin of higher transition temperatures obtained for the films prepared on other substrates, such as, i.e., 40 K observed in electrochemically etched ultrathin FeSe films grown on MgO (Shiogai et al., 2016). Moreover, the transition temperatures above 40 K have been also observed in iron selenide layers coated with metallic potassium (Miyata et al., 2015). The obtained *T*_c_ values are comparable to those found in bulk molecular intercalates of FeSe, which are described in more detail in the next few paragraphs. Another common feature of thin films and some intercalated iron selenides is a striking similarity of the Fermi surface topology, gap symmetry, and band structure, which indicates a similar mechanism standing behind superconductivity in these systems (see Figure 1) (Zhao et al., 2016). The similarities between the FeSe monolayers and bulk FeSe molecular intercalates point toward the possibility of further modification of these structures *via* the optimal selection of the type of intercalants, their stoichiometry, and ordering in the structure. As a result, it would be possible to obtain the heterostructures composed of Fe-Se monolayers separated by intercalating agents allowing for fine tuning of a coupling between the iron chalcogenide sheets, and thus achieving even higher transition temperatures to a superconducting state.

A specific layered structure, composed of infinite layers formed by edge-sharing FeSe_4_ tetrahedra, was arranged in stacks where the layers are held together by weak van der Waals interactions to make these materials very susceptible to intercalations with various chemical individuals, especially those that are capable to donate extra electrons to the iron selenide layers. On intercalation, the iron-selenide slabs are spaced apart by the intercession of an additional separation layer between them, and this process is usually accompanied by a slight structural transformation to a system of lower symmetry. In the case of intercalation with alkali metal ions, the iron-selenium layers, apart from the expected vertical spacing of the layers followed by a large expansion of the tetragonal unit cell, shift also horizontally nearly by 1/_2_*a*, allowing the intercalant to locate in the interplanar gap thus created. The intercalation is not quite topochemical as the structural modifications at the same time are observed (usually a change from primitive P*4/nmm* cell to body centered I*4/mmm* for alkali metal-intercalated iron selenides; Guo et al., 2010; Krzton-Maziopa et al., 2011; Wang et al., 2011), and to I*4/mmm* or P*4/mmm* when the bulk organic molecules are involved. The molecules of intercalant order in the van der Waals gaps creating infinite layers that may act as charge reservoirs or block the interactions between the individual FeCh_1−*x*_ slabs turning a whole system into the heterostructure consisting of monolayer-like slabs separated by sheets of intercalants. Interestingly, when larger intercalating moieties are incorporated into a van der Waals gap, the appearance of structural transition depends both on the type of molecular adduct formed with an alkali metal and the amount of intercalant between the layers of the parent material. Both of the abovementioned cases are extremely important to the superconducting properties of the parent compound, which captivating properties still remain unexplored and not fully understood.

According to the theoretical calculations, mechanical strains exerted either by isovalent substitutions in the anion sublattice of the parent iron selenides, the insertion of intercalants in the van der Waals gaps, or mechanically induced in thin films grown on piezoelectric substrates are essential to fine structural tuning and modulation of the band structure of layered iron chalcogenide-based superconductors (Winiarski et al., 2012; Paris et al., 2016; Guterding et al., 2017; Zhang et al., 2020). First-principle calculations have shown that significant modifications of the Fermi surface nesting can be induced even by a small *c*-axis strain, while the *ab*-plane strains suppress them considerably. Therefore, while considering future applications of these materials, one has to take into account these limitations, which possibly may lead to a partial deterioration of their properties. On the other hand, the mechanical stress, if well-controlled, can also serve as an additional tuning factor for the electronic properties of these materials (Zhou et al., 2019; Chaluvadi et al., 2021).

## Intercalated Iron Selenide—the Influence of the Chemical and Electrochemical Nature of Intercalating Species on the Crystal Structure

There are a few types of intercalating species that enter the structure of iron selenide and enhance *T*_c_ of the parent compound. Based on the available literature sources, these intercalants can be divided into three main groups: I—alkali and alkaline metal cations; II—solvates of selected alkali or alkaline earth metals with Lewis bases (usually ammonia, aliphatic amines, diamines, and aromatic amines); and III—alkylammonium cations. The properties of iron selenides intercalated with the three groups are briefly discussed below.

The first published report about iron selenide intercalated with alkali metal in 2010 evidenced that at elevated temperatures the polycrystalline FeSe_1−*x*_ annealed in potassium vapors changes its crystal structure as a result of the intercalation of alkali metal into the van der Waals gap (Guo et al., 2010). The layers of the inorganic host were pushed apart by potassium located in between the layers and finally a non-stoichiometric phase K_0.8_Fe_2−*x*_Se_2_, with an enhanced temperature to a superconducting state, reaching 30 K, was formed. Shortly after this first report, the Cs (Krzton-Maziopa et al., 2011) and Rb (Li et al., 2011; Wang et al., 2011) analogs were synthesized, followed by a multitude of compounds containing also mixed alkali metals in the structure, and monovalent thallium (Fang et al., 2011; Li et al., 2011). Depending on the chemical composition, all of these materials have shown transition temperatures to a superconducting state between 27 and 33 K (Tsurkan et al., 2011; Krzton-Maziopa et al., 2012a; Yan et al., 2012), accompanied by a significant expansion of the unit cell of the parent compound and the change of the unit cell symmetry from P*4/nmm* to I*4/mmm*. Several synthetic trials with a high-temperature approach have been also done using heavier alkaline earth metals, especially barium, but in the obtained materials only traces of superconductivity at much lower temperatures (around 12 K), in comparison to the systems containing monovalent cations were found.

**Figure 2:**
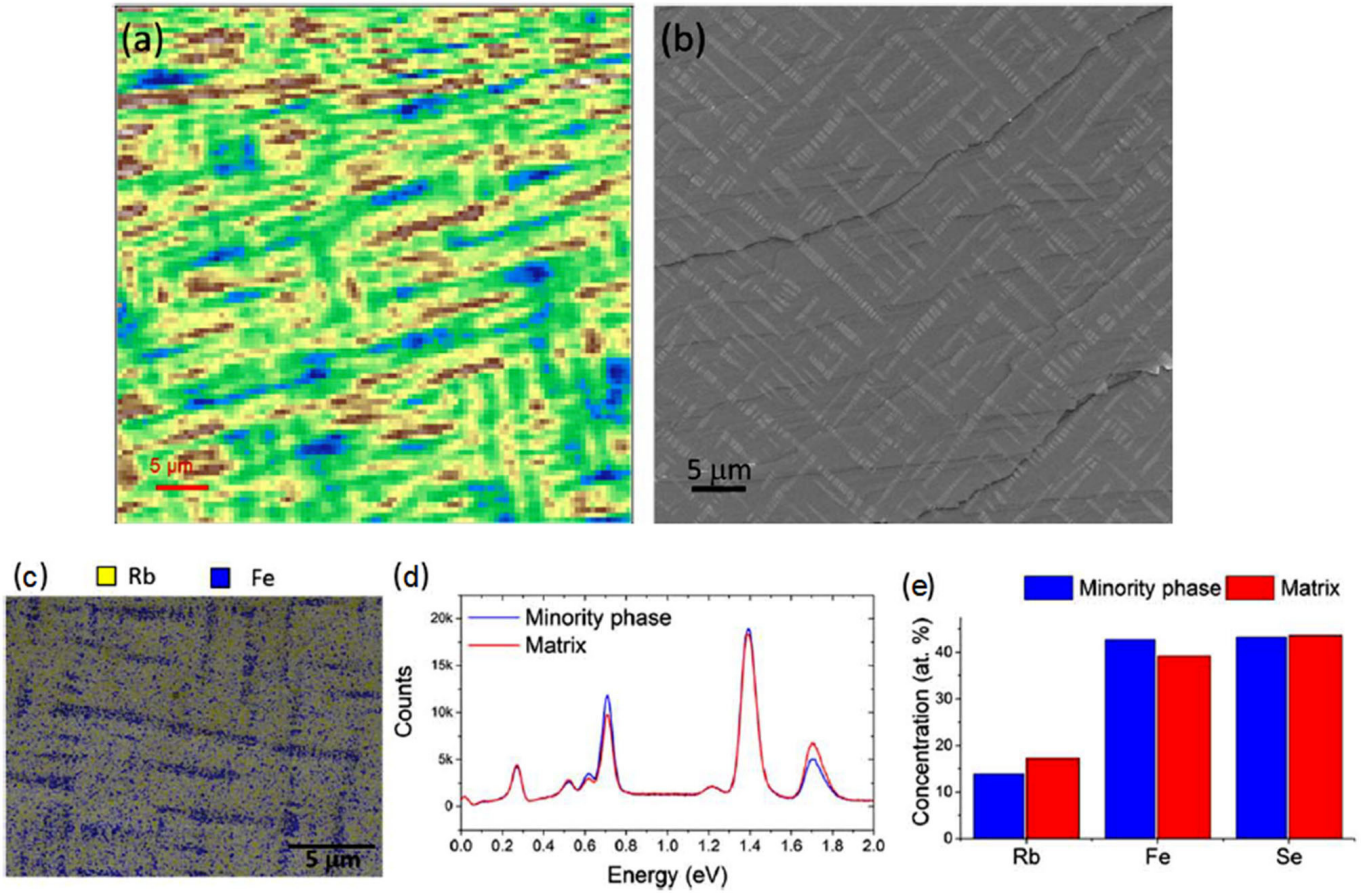
**(a)** Spatial map of valence band of Rb_*x*_Fe_2−*y*_Se_2_ crystal taken at the dynamic light scattering (DLS) NanoARPES end station with hν = 90 eV. **(b)** Scanning electron microscopy (SEM; secondary electron) image of a similar crystal. **(c)** Energy dispersive x-ray analysis (EDX) elemental maps taken at 10 kV. **(d)** EDX spectra reconstructed from minority phase and matrix regions of the sample. **(e)** Chemical compositions measured by EDX for each phase [Reprinted with permission from Dudin et al. (2019) Copyright (2019) IOP Publishing Ltd].

Alkali metals are strong reducing agents, with their formal redox potentials determined in the buffered neutral melt as low as −2.17, −2.18, −2.74, −2.80, and −2.90 V vs. NHE for Li, Na, K, Rb, and Cs, respectively (Scordilis-Kelley et al., 1992). Their high reducing ability plays a crucial role in the direct synthesis of A_*x*_Fe_2−*y*_Se_2_ from elemental iron and selenium, which is actually the most utilized route for the preparation of large crystals (Krzton-Maziopa et al., 2016). Elemental iron, which itself is also a good reducing agent, although weaker than all the alkali metals, serves as a *co*-reductant for nobler selenium in this reaction, which is carried out at high temperatures that additionally facilitate the reduction processes. The exact mechanism of the reaction nor its kinetics is known due to the technical difficulties of performing any measurements at temperatures above 1,300 K and a very aggressive environment of the vaporized alkali metal. The crystallization process is carried out at high temperatures, usually using vertical Bridgman or Bridgman–Stockbarger methods (Krzton-Maziopa et al., 2012a, 2016), which provide large crystals of A_*x*_Fe_2−*y*_Se_2_ (A = K, Rb, Cs, Rb/Tl, K/Tl) with the size limited only by the quartz ampoule dimensions. A high-temperature process cannot be treated as a true intercalation reaction because here all the reactants are melted and the new A_*x*_Fe_2−*y*_Se_2_ phase crystallizes directly from the melt and the final product is not obtained by the modification of the host structure by the insertion of foreign entities. Despite these inaccuracies in nomenclature, in the literature related to the iron selenide materials, this phase is usually called “intercalated” due to its structural analogy to the phases typically obtained in low-temperature solvothermal routes. Nevertheless, although not entirely correct from a chemical point of view, the term “intercalated iron selenides” became so entrenched in the minds of scientists dealing with these materials that it is practically impossible to eradicate them. Therefore, referring to the general nomenclature trend, this expression will be also used in the text while mentioning the A_*x*_Fe_2−*y*_Se_2_ phases obtained by conventional high-temperature synthesis. More details on the crystal growth and physical properties of the superconducting A_*x*_Fe_2−*x*_Se_2_ phases can be found in ample review papers (Chen and Lin, 2014; Chen et al., 2014; Johnson et al., 2015; Krzton-Maziopa et al., 2016). The most intriguing attributes of these materials are the presence of specific iron vacancy ordering, high Neel temperature, superconductivity accompanied by magnetic order, and still not fully explained is inherent microscale phase separation, visible in the form of small, several micrometer-sized plates arranged in a characteristic three-dimensional network of interconnected paths percolating through the entire crystal, visible in crystallites of all 122-intercalated phases grown directly from the melts (Speller et al., 2012; Liu et al., 2016). The illustration of the phase separated material is shown in Figure 2. The size of the minority phase domains that are composed of small plates arranged along the {113} habitus plane and twisted about an angle of 30°, is of the order of few micrometers.

Despite the observation of phase separation in A_1−*x*_Fe_2_Se_2_, the transport, magnetization, and heat capacity studies confirmed 100% Meissner shielding in these materials, which seems a bit controversial in the context of a phase separation phenomenon evidenced so far by neutron diffraction (Yu Pomjakushin et al., 2012; Zhao et al., 2012), x-ray diffraction and spectroscopy studies (Ricci et al., 2011; Bosak et al., 2012; Svitlyk et al., 2018), muon spin rotation spectroscopy (Charnukha et al., 2012; Shermadini et al., 2012), NMR (Torchetti et al., 2011; Texier et al., 2012), scanning electron microscopy (SEM) (Speller et al., 2012, 2014), scanning tunneling microscopy (STM) (Ding et al., 2013), and magnetic force microscopy (MFM) imaging (Hazi et al., 2018; Dudin et al., 2019). After nearly a decade of research on the origin of phase separation phenomena, there is a general agreement that the vacancy-free A_1−*x*_Fe_2_Se_2_ superconducting phase forms only in the presence of an insulating vacancy-ordered antiferromagnetic majority phase with the chemical composition close to A_0.8_Fe_1.6_Se_2_ (Speller et al., 2012; Carr et al., 2014; Bao, 2015) while the superconducting one is a metallic vacancy-free phase. There were also several structural models suggested for both these phases involving the different ordering of iron and alkali metal vacancies, specific ordering of charges and spins, and magnetic domains orientation (Li et al., 2012; Ding et al., 2013; Carr et al., 2014; Svitlyk et al., 2018), but until today any consistent model covering all the experimental observables has not been proposed. It has been experimentally proved that tiny domains of the minority phase are uniformly distributed in the AFM-ordered majority A_0.8_Fe_1.6_Se_2_ phase, forming a specific percolation network throughout the whole macroscopic crystallite, enabling high Meissner screening. The phase separation effect in these materials turns out to be reversible in a relatively narrow temperature range and annealing the crystals at the temperature corresponding to the phase separation, below the temperature of the iron vacancies ordering, causes a microscopic reorganization of plates of the minority phase resulting in superconducting properties of these materials (Bosak et al., 2012; Weyeneth et al., 2012; Yu Pomjakushin et al., 2012; Speller et al., 2014; Svitlyk et al., 2018). Moreover, from the available experimental data, it appears that the temperature range of the phase transitions depends primarily on the concentration of the alkali metal and not on the ionic radius of the intercalating ion. The minority phase is assumed as iron vacancy free with the chemical composition A_*x*_Fe_2_Se_2_ indicating the complete occupation of the iron sublattice and the presence of a considerable amount of alkali metal vacancies in the structure (Porter et al., 2015). The determination of the exact amount of an alkali metal present in the minority phase is strong “technique dependent” and ranges from 0.6 (according to neutron diffraction studies) to about 0.3 based on the NMR results (Yu Pomjakushin et al., 2012; Fang et al., 2013). Oxidation state of iron ions in the minority phase was found to be slightly lower than +2, suggesting that the electron doping is a main factor governing the superconducting behavior (Basca et al., 2011; Texier et al., 2012), which should be considered while designing novel intercalated systems based on layered iron chalcogenides.

**Figure 3:**
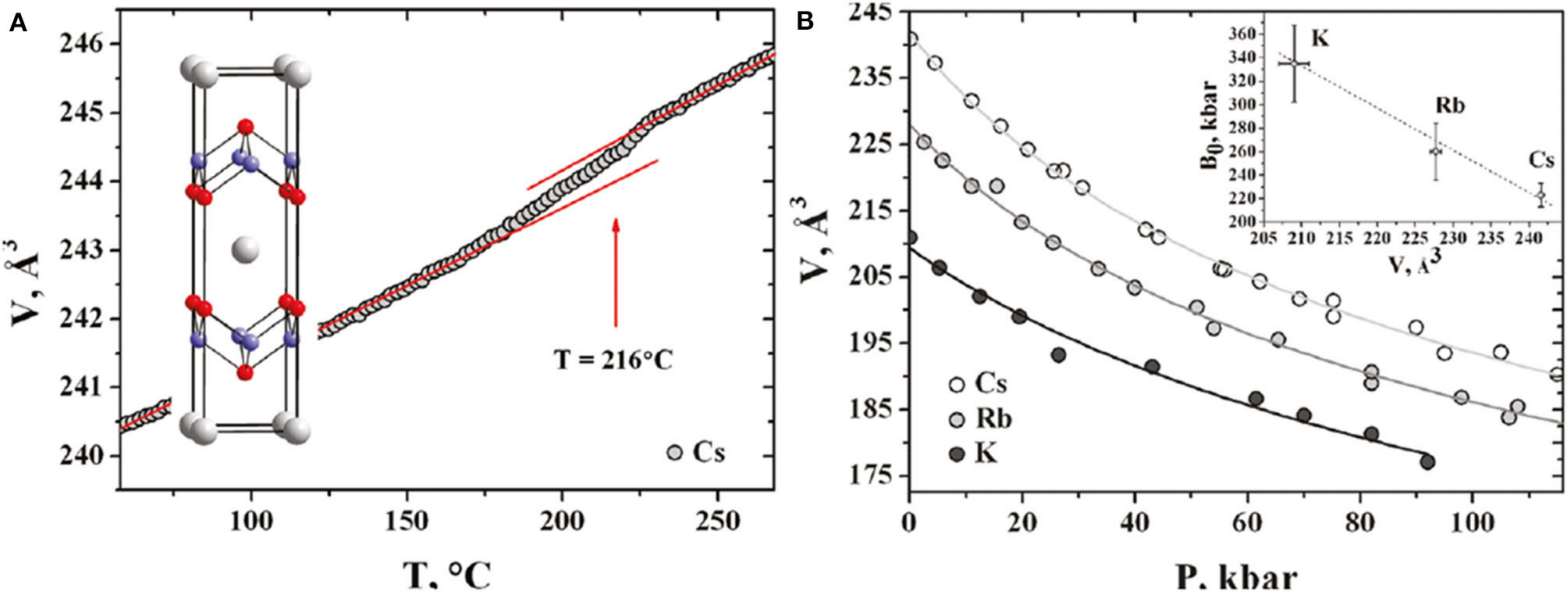
**(A)** Temperature-dependent first-order *I*4/*m* to *I*4/*mmm* structural transformation measured by synchrotron powder diffraction for Cs_*x*_Fe_2−*y*_Se_2_. **(B)** Volume vs. pressure dependences for Cs, Rb, and K intercalated crystals fitted with the Murnaghan equation of state. Inset shows bulk moduli vs. the unit cell volumes at ambient pressure [Reprinted with permission from Svitlyk et al. (2011) Copyright (2011) American Chemical Society].

The unusual coexistence of superconductivity and magnetic ordering with enormously high Neel temperature present in these materials is a direct evidence for the presence of the long-range antiferromagnetic ordering. Neutron studies confirmed a direct relationship between the crystal structure and magnetic parameters and revealed reasons of the presence of magnetic ordering in alkali metal-intercalated iron selenides. High-pressure transport studies of Cs_*x*_Fe_2−*y*_Se_2_ crystals showed almost constant *T*_c_ up to 5 GPa, followed by a sharp decrease above 8 GPa followed by its complete suppression accompanied by a partial degradation of the material at high pressures. Detailed investigation of the effect of temperature on the Cs_0.83_Fe_1.72_Se_2_ structure with synchrotron radiation (shown in Figure 3) confirmed the first-order phase transition (*I*4/*m* to *I*4*/mmm*) at about 490 K associated with disordered iron vacancies in the material. Partial irreversibility of this transformation is most probably the result of enhanced mobility of alkali metal ions in the structure at this temperature. Studies on rubidium-intercalated Rb_*x*_Fe_2−*y*_Se_2_ crystals confirmed the existence of similar dependence as a function of external pressure illustrated in Figure 3B. In turn, based on the study of the influence of external pressure on the crystal structure of Cs_0.83_Fe_1.72_Se_2_, Rb_0.85_Fe_1.72_Se_2_, and K_0.8_Fe_1.72_Se_2_, it was found that reflections, characteristic for *I*4*/m* symmetry, derived from the superstructure of ordered vacancies, are visible even at a high pressure of 12 GPa (Svitlyk et al., 2011). This confirms the existence of a specific arrangement of iron vacancies in these materials both in the superconducting state and the normal state.

Alkali metal-intercalated superconducting selenides hide their secrets very effectively, without letting them be investigated in detail. It should be emphasized that these materials are easily degraded in contact with oxygen and moisture, additional heating with radiation beams may also cause local changes in the chemical composition due to the enhanced mobility of the alkali metals or even their partial deintercalation in the surface region. Moreover, the content of the superconducting phase in A_*x*_Fe_2−*y*_Se_2_ crystals, is usually smaller than 12% by volume (Bosak et al., 2012; Shermadini et al., 2012; Speller et al., 2012; Yu Pomjakushin et al., 2012), and the minority phase plates are typically smaller than the diameter of the beams with which they are investigated, which causes additional difficulties with a precise determination of the chemical composition and some discrepancies in the results obtained by various research groups. The development of nanosized beams for investigations improved the situation a little bit (Ricci et al., 2011; Hazi et al., 2018; Dudin et al., 2019; Chen et al., 2021), allowing for imaging of the electronic structure of superconducting phases but still the same questions related to the origins of superconductivity and its coexistence with antiferromagnetically ordered phase remain unanswered, raising the need for further investigation of these peculiar materials. Studies on the thermal behavior of A_*x*_Fe_2−*y*_Se_2_ crystals using calorimetric methods combined with neutron diffraction, microscopic imaging, and magnetic studies (Weyeneth et al., 2012; Yu Pomjakushin et al., 2012; Speller et al., 2014) revealed the characteristic temperatures for phase transitions and a mesoscale phase separation in these materials indicating the need for research on alternative low-temperature synthesis pathways allowing for the preparation of materials free from phase separation. The answer came soon with the first reports on the discovery of superconductivity in polycrystalline materials prepared by solvothermal intercalations of layered iron selenides in solutions of selected alkali metals in liquid ammonia (Ying et al., 2012) and in heterocyclic pyridine (Krzton-Maziopa et al., 2012b). Solubilization of some alkali metals (Li, Na, and K), alkaline earth metals (Ba, Sr, and Ca), and rare earth metals (Yb and Eu) in liquid ammonia, or lighter alkali metals (Li, Na, and K) in pyridine and utilization of these solutions as intercalating agents of II type (the solvates of alkali/alkaline earth metals with Lewis bases), enabled for the preparation of the first superconducting iron selenides with cointercalated solvent molecules with transition temperatures to a superconducting state reaching 46 K (Krzton-Maziopa et al., 2012b; Ying et al., 2012). The superconducting volume fractions estimated from the magnetization studies of the as-prepared ammonia-containing compounds were found to be between 5% for Yb and 62% for Ba, for lighter alkali metals (Li and Na) they were close to 30%, which anyhow was higher than in the materials grown by a conventional high temperature method. An interesting behavior was found in pyridine-containing intercalates, where for the as-prepared materials only a weak signal around 45 K was detected in magnetization studies, whereas the resistivity measurements have shown a clear transition to the superconducting state starting around 46 K with an additional step around 31 K indicating the presence of two phases differing in superconducting properties. This two-step transition was a clear suggestion that the intercalated organic molecules are most probably disordered in the structure. Annealing of the prepared materials in the sealed ampoules greatly improved the superconducting response, and the shielding fraction was enhanced nearly to 80% (Krzton-Maziopa et al., 2012b; Biswas et al., 2013; Komedera et al., 2016). A great improvement of shielding fraction in the pyridine-based intercalate resulted most probably from the reorganization of a molecular intercalant in the van der Waals gap of the parent iron selenide. The ordering of organic molecules in the structure was clearly evidenced by a structural transition from *I4/mmm* (No. 139) to a larger supercell with *P*4*/mmm* (No. 123). The exact structural model for these materials is still not fully developed due to the relatively poor crystallinity of pyridine intercalates. The most detailed structural studies up to now were performed on ammonia-containing materials. The experimental efforts put on the improvement of a synthetic approach as well as the preparation of deuterated materials allowed for modeling of the crystal structure of these materials and the studies on their superconducting behavior in ammonia-rich and ammonia-deficient systems (Scheidt et al., 2012; Burrard-Lucas et al., 2013).

The solvothermal intercalation of iron selenide in liquid ammonia is based on the controlled introduction of the intercalant at low temperature into the layered structure of this compound. The solid polycrystalline selenide, along with the appropriate amount of lithium (or other alkali metal), is placed in the Schlenk reactor under an inert gas atmosphere (usually high purity argon). The reactor is connected to a vacuum line where the gas is evacuated, and the reactor is cooled most often in liquid nitrogen or dry ice to the temperature below the ammonia condensation temperature (239.82 K). At the same time, gaseous ammonia is introduced into the system, which at low temperature condenses on the reactants and dissolves the alkali metal, creating immediately a dark blue solution in which the polycrystalline selenide is immersed. After a desired duration of the intercalation process (from tens of minutes to few 100 h, depending on the solubility of a metal used in the process), ammonia is slowly removed from the reaction medium by heating the reactor to room temperature. The concentration of metal dissolved in liquid ammonia in these intercalations is typically set 0.1–0.3 mol/dm^3^. The intercalation process for larger amines is a bit longer due to the weaker solubility of metals in amines and higher temperatures are needed for the intercalants to enter the structure of the inorganic host due to a bulkier structure of these organic molecules. In case of amines, the concentration of about 0.2 mol/dm^3^ of the dissolved metal is used. The products after the intercalation have a similar appearance—the volume of a very fine black solid powder after drying increases at least twice, depending on the type of amine and the type and concentration of the metal used in the process.

The products are extremely air-sensitive and moisture-sensitive due to the presence of alkali metals in the structure and a highly developed specific surface area, therefore any contact with atmosphere or humidity must be avoided, otherwise, they immediately decompose. Through proper adjustment of the molar ratio of reagents and other conditions of the intercalation process, one can obtain materials of high superconducting volume fraction with *T*_c_ = 46 K. In case of ammonia-based intercalates, the highest purity of materials (the lowest content of magnetic impurities) was obtained at molar ratios of alkali metal and iron selenide close to 1:2 (Scheidt et al., 2012; Burrard-Lucas et al., 2013; Zheng et al., 2013; Lee et al., 2018). Ammonia-based and amine-based intercalates do not seem to be very pure systems as in the final products, depending on the concentration of alkali metal in the solvent, various types of solvates with metal ions are formed, which may concomitantly enter to the structure. This effect was already observed in the first reports about ammonia-based materials, as the exact stoichiometry of the intercalant was not clear. There was a hot debate raising the probability of coexistence of different types of alkali metal-solvent adducts, i.e., Li_*x*_(NH_3_)_*y*_, or even the possibility of formation of alkali metal amides (LiNH_2_) or a mixture thereof located between the iron-selenium layers (Burrard-Lucas et al., 2013). Structural studies performed *in situ* on the deuterated ammonia-containing intercalates proved beyond doubt that both of these chemical entities enter the structure of the matrix compound in the intercalation process (Sedlmaier et al., 2014). Intercalation with lighter alkali metals in liquid ammonia is a relatively fast process and the product contains the most thermodynamically stable single phase. Intermediate phases immediately transform and do not exist long enough to be detected even with *in situ* studies, but it is possible while using solutions of alkaline earth metals as intercalating agents. Yusenko et al. investigated the phase evolution in Ba-ammonia intercalates (Yusenko et al., 2015). It was found that by varying the conditions and concentration of ammonia in the system, one can tune both the crystal structure and superconducting properties. Among all the detected phases ([Ba_0.29_(NH_3_)_0.35_Fe_2_Se_2_] *I*4/*mmm*; [Ba_0.24_(NH_3_)_1.92_Fe_2_Se_2_] *I*4/*mmm, T*_c_ = 39 K; [Ba_0.35_(NH_3_)_2.12_Fe_2_Se_2_] *P*4/*nmm*; [Ba_0.37_(NH_3_)_1.04_Fe_2_Se_2_] *P*4/*nmm, T*_c_ = 36 K; [Ba_0.28_Fe_2_Se_2_] *I*4/*mmm, T*_c_ = 34 K) only three were superconducting, and the highest critical temperature (39 K) was measured for an ammonia-rich sample of limited stability controlled by the availability of liquid solvent. This raises the questions about the stability of molecular intercalates containing volatile solvents in solvent poor environments, which is crucial when small amounts of intercalated powders are handled in open space letting the volatile organic compound to be released. The usage of less volatile media (i.e., long-chain amines and heterocycles of higher boiling points than ammonia) for the intercalation can partially solve this problem but at the same time, new ones related to the formation of metastable phases with no superconducting properties may arise. Moreover, the intercalation process is diffusion controlled, and the diffusion coefficients for bulkier species are much smaller in comparison to the others. Investigations on various amine-based systems [pyridine (Krzton-Maziopa et al., 2012b), aliphatic diamines: ethylenediamine (Noji et al., 2014; Majcen Hrovat et al., 2015; Gao et al., 2018), propanediamine and butanediamine (Jin et al., 2017), or hexamethylenediamine (Hosono et al., 2014, 2016)] revealed the possibilities of further structural modifications in these systems leading to the preparation of materials with enhanced stability regarding to the release of cointercalated solvent molecules, although the reactions are more time consuming and usually last from 1 day to a couple of weeks when a more viscous solvent is used (Hosono et al., 2014). Amine solutions of alkali metals, similarly to solutions of alkali metals in ammonia, are very strong reducing agents, therefore a partial chemical reduction of the matrix occurs during this reaction. Shorter contact times of the matrix with the reducing agent is preferred due to the possibility of the formation of unwanted side products coming both from the degradation of an intercalating medium (i.e., bond cleavage, ring opening, dimerization/oligomerization of amines, and the formation of alkali metal amides) and excision of selenium or iron from the inorganic hosts leading to the formation of alkali metal selenides, and the occlusion of small clusters of elemental iron in the material. The process parameters still need to be optimized for all the amines investigated so far. Another problem that also needs further attention is the progressive delamination of the host and deteriorating crystallinity of the material with the length of the solvothermal intercalation process. Although the usage of alkali metals coordinated by Lewis bases for the intercalation requires a prolonged time of the process and the product is frequently contaminated by magnetic impurities, this approach enables the preparation of intercalates in which the organic molecules stay in the structure, and they do not deintercalate even when the material is exposed to air. Moreover, the expanded crystal structure of such an intercalate is preserved, which makes it possible to use them in catalysis or in the construction of systems for energy generation, storage, and conversion (Jin et al., 2017). The structure retention opens also an excellent opportunity to prepare new hybrid materials through the ligand exchange. On the other hand, it has been shown recently that by appropriate synthesis conditions it is possible to tune the linkage between iron selenide tetrahedra and directly grow FeSe derivatives exhibiting superconductivity and a rich variety of magnetic behavior ranging from antiferromagnetism to paramagnetism (Pak et al., 2013; Greenfield et al., 2015; Stahl et al., 2018).

**Figure 4:**
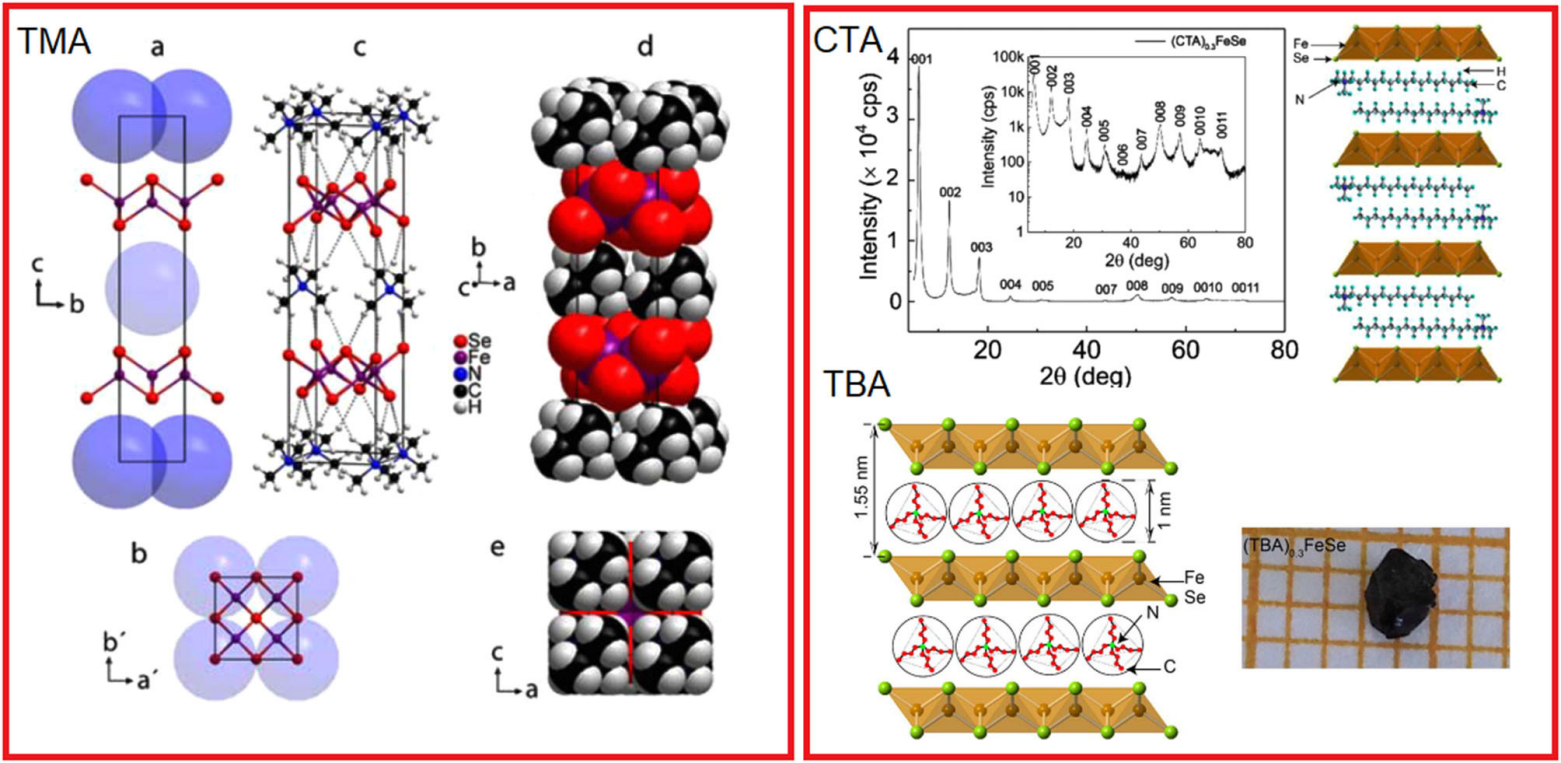
Structural models of three alkali metal-free materials obtained by electrochemical intercalation. Left panel: structural model of tetramethylammonium- (TMA-) intercalated FeSe **(a)**, doubled unit cell **(b)**, full structural model of (TMA)_0.5_Fe_2_Se_2_ in the space group *I42m* with hydrogen bridges shown as dashed bonds **(c)**, space-filling model **(d)** and view perpendicular to the TMA+ layers **(e)**. Right panel: structural models proposed for intercalation containing cetyltrimethylammonium (CTA) and tetrabutylammonium (TBA) cations [Reprinted with permission from Rendenbach et al. (2021) and Shi et al. (2018)].

The third but no less important group of intercalants are alkylammonium ions inserted into the structure of iron selenide by electrochemical methods. Here, the intercalation process is alkali metal free, and the chalcogenide matrix is reduced in an electrochemical intercalation process. Currently, three materials intercalated with the following cations such as cetyltrimethylammonium (CTA) (Shi et al., 2018) with *T*_c_ = 45 K, tetrabutylammonium (TBA) with record high *T*_c_ = 50 K, and the third one intercalated with tetramethylammonium (TMA) (Rendenbach et al., 2021) showing *T*_c_ = 43 K, obtained by this method are known. All of them are characterized by critical temperatures comparable to iron selenides intercalated with ammonia/amine solvates of alkali metals, and the presence of organic cations only between the layers of the inorganic host. According to the source material, the (CTA)_0.3_FeSe has a hybrid crystal structure consisting of double layers of CTA^+^ separated by a single FeSe slab. Structural models proposed for these materials are shown in Figure 4. Like other molecular intercalates, this material also exhibits a negative pressure effect on superconductivity d*T*_c_ /d*P* = −5 K/GPa (Sun et al., 2020). An electrochemical approach offers a unique opportunity for the synthesis of monocrystals enabling detailed studies of the crystal structure of intercalated materials. Although some time already passed, the exact models for crystal structures for CT and CBA materials have not yet been provided, most probably due to a large disorder of the organic entities intercalated in the structure. This was, however, done with a high accuracy for a TMS-intercalated material, which significantly broadens the knowledge in chemistry of these materials. TMS-intercalated FeSe crystallizes in the tetragonal ThCr_2_Si_2_ structure (space group *I*4/*mmm*) with a large expansion of *c*-lattice parameter *c* = 20.377 (3) Å and *a* = 3.8585 (2) Å. The as-prepared material is claimed to show large shielding close to 100% although accompanied by a small ferromagnetic contribution of unknown origin.

As it was shown above, a variety of intercalants can be accommodated in the layered structure of iron selenide modifying its superconducting and magnetic properties. Looking closer to the systems intercalated with alkali metals and with their solvates, one can see some chemical similarities: in both cases the intercalant enters the structure and reduces partially the parent compound. In the high-temperature route, the reaction leads to the chemical reduction of a part of Fe ions in FeSe to elemental iron that precipitates from the melt during slow cooling and usually locates in the tip of the crystals grown by vertical Bridgman methods (Krzton-Maziopa et al., 2012b). This Fe-enriched tip of the crystal is not superconducting and contains inclusions of large irregular clusters of the iron-rich phase. More interestingly, the crystals obtained by the rapid quenching of the melts from high temperature also show the presence of iron clusters distributed in the whole volume of the obtained crystals, and their superconducting response, if present, is very small. The second type of intercalating agents is less aggressive to the iron selenide matrix, although also of very high reduction ability provided by the presence of solvated electrons in the system that is delocalized to the solvent on solubilization of alkali metals (Thompson, 1976). From the chemical point of view in the system where solvated electrons are generated, both the solvent and metal that dissolves in it have comparable affinities to the electron, which in such cases is located in cavities formed by the molecules of solvents and counter ions (Shkrob, 2006; Zurek et al., 2009; Chaban and Prezhdo, 2016). In liquid ammonia, solvated electrons usually form large clusters of about 5 Å in size. Both alkali and alkaline earth metals are easily ionized and strongly solvated by ammonia molecules. Alkali metals typically coordinate four ammonia molecules, while alkali earths are surrounded by six of them in their first coordination sphere (Chaban and Prezhdo, 2016). The experimental evidence of the formation of solvated electrons in diluted solutions of quite few metals in ammonia is the similarity of their absorption spectra with a characteristic tail (Blades and Hodgins, 1955; Zurek et al., 2009). At lower concentrations of 0.01–0.04 molar fraction of the metal, all their solutions in anhydrous ammonia have a dark blue color. At higher metal concentrations, the solution turns into a very conducting liquid up to the saturation (about 0.2 molar fraction of a metal), then rapidly changes into golden liquid resembling a low viscous metallic melt. Depending on the concentration of metal dissolved in ammonia or amine, different types of aggregates, ranging from solvated entities to coupled radicals, can be formed (Catterall and Symons, 1965). All of them may enter the van der Waals gap of the parent compound, but their electric and magnetic properties are different and may influence considerably the superconducting properties of the whole system. In the process of formation of these entities, the free enthalpy of solvation of the metal is a crucial and limiting factor determining the solubilization process and the electron transfer to the solvent (Fowles et al., 1957; Bar-Ali and Tuttle, 1966). Ammonia or short-chain primary amines are considered as small molecules with lower enthalpies of solvation and are favored to form concentrated solutions of alkali metals. A similar behavior was found also in diamines (Zurek, 2011) and aromatic pyridine (Schmulbach et al., 1968). On the search for other molecules that may be intercalated between the layers of iron selenide, it was confirmed that the insertion of other donors such as LiFeO_2_ or [(Li_1−*x*_Fe_*x*_)OH] is also possible, resulting in *T*_c_ = 40 K, though accompanied by a ferromagnetic contribution visible in a low-temperature region (Lu et al., 2014; Pachmayr et al., 2015; Sun et al., 2015). Interestingly, the authors of the first report on these oxide intercalates initially mistakenly suggested that it was magnesium oxide that incorporates into the structure of iron selenide (Lu et al., 2013). Then, they proposed that the phase is LiFeO_2_Fe_2_Se_2_ (Lu et al., 2014), but subsequently established, along with other groups (Pachmayr et al., 2015; Sun et al., 2015) that the compounds are in fact hydroxide intercalates (Lu et al., 2015). The advantage of those oxide-intercalated and hydroxide-intercalated materials is their much better stability in the air in comparison to ammonia-/amine-based systems. Two of the mentioned groups of intercalants have been already tried as intercalating guests for ternary iron selenides with variable effects, but none of these materials investigated so far was better than those described above.

## Conclusions and Future Directions

Enormous interest in non-stoichiometric transition metal chalcogenides was stimulated 10 years ago with a discovery of superconductivity in the tetragonal phase of iron monoselenide (Mizuguchi et al., 2008). Still after 10 years, the subject attracts the interest of researchers due to new possibilities offered by this system. As it was shown above, three groups of intercalating moieties were already embedded into the layered structure of iron selenides. The structure seems to be capable to accept even larger spacers enabling for the preparation of hybrid materials for different applications that are becoming more and more visible. Thin films of nanocrystalline iron selenides have been already tested as absorbing layers in photovoltaic devices and also as a potential replacement for expensive platinum in dye-sensitized photovoltaic cells (Ubale et al., 2013). Moreover, due to the possibility of a reversible intercalation with alkali metal ions and a high diffusion coefficient associated with this process (~10^−6^ cm/s), iron selenide was also tested as a potential electrolyte for use in, e.g., lithium batteries (Jiang et al., 2016). Despite the controversies on the sources of superconducting properties, inaccuracies in the actual superconducting phase stoichiometry and phase separation phenomena that additionally complicate the whole system suppressing unambiguous determination of factors governing superconducting properties, the layered iron-based superconductors have been the most investigated materials in the field of condensed matter physics and materials science over the last decade and still did not reveal all their secrets.

Undoubtedly, the molecular intercalates of iron selenide possess superior superconducting properties, that are strongly enhanced by the dimensionality and electronic polarizability of intercalating guests. It can be roughly assumed that all three types of described intercalants work on the similar principle. The chalcogenide matrix is reduced either by the action of the alkali metal that enters its structure, or electrochemically, and the intercalant plays a role of charge reservoir and a separator blocking the interactions between the layers. The solvothermal/electrochemical approach enables the preparation of single-phase materials that were not possible with high-temperature crystallization processes. Although much remains to be done in the area of refining the conditions of synthesis, improving crystallinity, optimizing doping, understanding the mechanisms of superconductivity, this approach makes it possible to obtain the materials containing nearly 100% of the superconducting phase. The discovery of such possibilities is a major step toward an understanding of the origin of superconducting properties in these fascinating materials. This raises the question about the future development in the research of these novels and yet still not fully understood materials. Certainly, the work on the hybrid systems is still at the beginning and needs more systematic research both on the synthesis and a detailed structural characterization of novel phases, involving mutual interactions between an intercalant and a host phase, and driving forces for a specific orientation of intercalant molecules in the van der Waals gaps of the parent compound. Clarification of the character of these interactions will be particularly supportive in future explanations of mechanisms standing behind the superconducting and magnetic properties of organic–inorganic hybrids. Bearing in mind the most unexplored areas, future research on the intercalated systems will focus on the explanation of the relation between structural changes, involving chemical pressure effects and optimization of crystal structure geometry. The second direction will be associated to modifications of the electrical and magnetic properties of hybrid materials by the alteration of electron doping and tuning the band structure. Moreover, as the hybrid materials developed so far are characterized by limited stability being the result of volatile solvents inserted in the structure, the material development will focus on a search for intercalants with a greater affinity to Fe-*Ch* host layers and improved environmental stability.

## Author Contributions

The author confirms being the sole contributor of this work and has approved it for publication.

## Conflict of Interest

The author declares that the research was conducted in the absence of any commercial or financial relationships that could be construed as a potential conflict of interest.
